# The Effect of Maternal Diet and Physical Activity on the Epigenome of the Offspring

**DOI:** 10.3390/genes15010076

**Published:** 2024-01-06

**Authors:** Anastasia Panagiotidou, Christos Chatzakis, Athina Ververi, Makarios Eleftheriades, Alexandros Sotiriadis

**Affiliations:** 1 School of Medicine, Aristotle University of Thessaloniki, 546 22 Thessaloniki, Greece; anastasia_pngtd@hotmail.com (A.P.); cchatzakis@gmail.com (C.C.); info@athinaververi.org (A.V.); 2 Second Department of Obstetrics and Gynecology, School of Medicine, Aristotle University of Thessaloniki, 546 22 Thessaloniki, Greece; 3 Genetic Unit, First Department of Obstetrics and Gynecology, School of Medicine, Aristotle University of Thessaloniki, “Papageorgiou” General Hospital, 564 03 Thessaloniki, Greece; 4 Second Department of Obstetrics and Gynecology, National and Kapodistrian University of Athens, 115 28 Athens, Greece; makarios@hotmail.co.uk

**Keywords:** maternal factors, pregnancy, diet, physical activity, epigenetic modifications, DNA methylation, miRNA, offspring

## Abstract

The aim of this review was to examine the current literature regarding the effect of maternal lifestyle interventions (i.e., diet and physical activity) on the epigenome of the offspring. PubMed, Scopus and Cochrane-CENTRAL were screened until 8 July 2023. Only randomized controlled trials (RCTs) where a lifestyle intervention was compared to no intervention (standard care) were included. Outcome variables included DNA methylation, miRNA expression, and histone modifications. A qualitative approach was used for the consideration of the studies’ results. Seven studies and 1765 mother–child pairs were assessed. The most common types of intervention were dietary advice, physical activity, and following a specific diet (olive oil). The included studies correlated the lifestyle and physical activity intervention in pregnancy to genome-wide or gene-specific differential methylation and miRNA expression in the cord blood or the placenta. An intervention of diet and physical activity in pregnancy was found to be associated with slight changes in the epigenome (DNA methylation and miRNA expression) in fetal tissues. The regions involved were related to adiposity, metabolic processes, type 2 diabetes, birth weight, or growth. However, not all studies showed significant differences in DNA methylation. Further studies with similar parameters are needed to have robust and comparable results and determine the biological role of such modifications.

## 1. Introduction

Although genotype is a major determinant of a person’s trajectory, lifestyle and environment can also influence health and well-being. The intrauterine environment and maternal exposure to exogenous (diet and physical activity, pollutants, toxic agents, smoking, and infections), and endogenous factors [maternal obesity, gestational diabetes mellitus (GDM), stress, and endocrine disruptors] can strongly influence the growth of the embryo, induce adverse pregnancy outcomes [1,2], and even cause predisposition for late-onset morbidity [3,4,5,6]. The environmental influence on the fetus is a process known as fetal programming [7,8] and is considered to “prepare” the fetus for the conditions of the outside world [5,9].

Epigenetic modifications are considered a possible mechanism by which maternal lifestyle affects the health of her offspring. Epigenetic modifications can alter gene expression without interfering with DNA sequence, just by regulating chromatin structure and organization, or mRNA expression [5,10]. The main types of epigenetic modifications are DNA methylation, histone modifications, and the expression of micro-RNAs (miRNAs); these mechanisms interfere with each other, and some can be passed to the next cell generation or even through the germline [10].

DNA methylation (DNAm) occurs when a methyl group (CH3) is added to a cytosine in CpG dinucleotides. Methylation at promoters is usually linked with gene repression [11], while methylation in the gene’s body is associated with gene expression [12]. Mainly, DNA methylation maintains a repressed transcriptional status in genes [13]. A global genome demethylation occurs just after fertilization, while maintaining the allele-specific methylation of imprinted genes [14,15], and a re-methylation follows in the blastocyst stage. Among samples, tissues, or persons, regions with different methylation status, called differentially methylated regions (DMR) or dmCpGs, can be found and seem to be associated with gene regulation. The identification of such regions among samples implies epigenetic differences [16]. Histone modifications also regulate chromatin structure and organization allowing transcription factors and other proteins to bind to the DNA [17]. Lastly, miRNAs are small non-coding RNAs that bind onto transcribed mRNAs and inhibit their expression [13,18]. miRNAs can cross the placental barrier and be transferred between mother and fetus [19].

Many studies have already associated several conditions in pregnancy such as GDM [20,21,22,23], or increased maternal BMI [24,25,26,27,28], maternal diet [29,30,31,32,33,34], pollutants [35,36,37,38,39,40], or stress [41,42] with the offspring’s methylation. However, there are limited reviews that gather and combine the existing knowledge on the topic. The aim of this review was to summarize and consider the current literature regarding the contribution of maternal lifestyle interventions (diet and physical activity) to the offspring’s epigenome. We intended to investigate the direct effect of interventions on the examined outcome; therefore, we only included randomized controlled trials (RCTs) and no other types of studies.

## 2. Materials and Methods

This systematic review was created according to PRISMA protocols and is registered with PROSPERO (CRD42023463422).

### 2.1. Eligibility Criteria

We investigated only RCTs in which a lifestyle intervention during pregnancy was compared to no intervention. The intervention included dietary advice or physical activity, and no intervention included standard care in pregnancy. The outcomes examined were DNAm, miRNA expression, and histone modifications. Only articles in English were assessed, but no restrictions in other parameters were imposed. Only maternal lifestyle modifications affecting the epigenome of the offspring were examined. Sperm-/paternal lifestyle-related studies were excluded, as well as animal models and studies using supplements. However, studies in which the intake of a particular substance was related to a specific lifestyle or diet, e.g., Mediterranean Diet, were considered relevant and were retained.

### 2.2. Information Sources and Search Strategy

A systematic search in three databases (PubMed, Scopus, and Cochrane-CENTRAL) was conducted on 8 July 2023. The search used both keywords and word variants of the terms “periconceptional”, “pregnancy”, “maternal”, “offspring”, “infant”, “DNA methylation”, “miRNA”, “epigenetic modifications”, “histone acetylation”, “lifestyle”, “diet”, “physical activity”, “sedentary”, and “stress”. A detailed example of the search strategy is shown in Appendix A.

### 2.3. Study Selection and Data Extraction

Data were evaluated using a customized data extraction form that included authors’ names, publication date, type of study, characteristics of the participants, intervention, intervention period, recorded outcome, and results of the studies. A PICO Table was constructed to summarize information from the primary articles.

Data extraction and risk assessment were conducted by two authors (AP and CC). Discrepancies were resolved by a third author (AV or AS). Mendeley was used in the article selection and duplicate elimination process.

### 2.4. Risk Assessment

The risk of bias of individual studies was estimated using the Revised Cochrane Risk-Of-Bias Tool for Randomized Trials (RoB 2) [43]. The following were evaluated: bias in randomization process, bias due to deviations from the intended interventions or missing outcome data, and bias in the measurement of the outcome or selection of the reported result. Then, the included articles were considered as having high/low overall risk of bias or some concerns depending on the number of individual risk parameters in each of the sections.

In order to identify the study characteristics, PICO was used. PICO is presented in Table 1 below.

## 3. Results

### 3.1. Search Results

**Figure 1:**
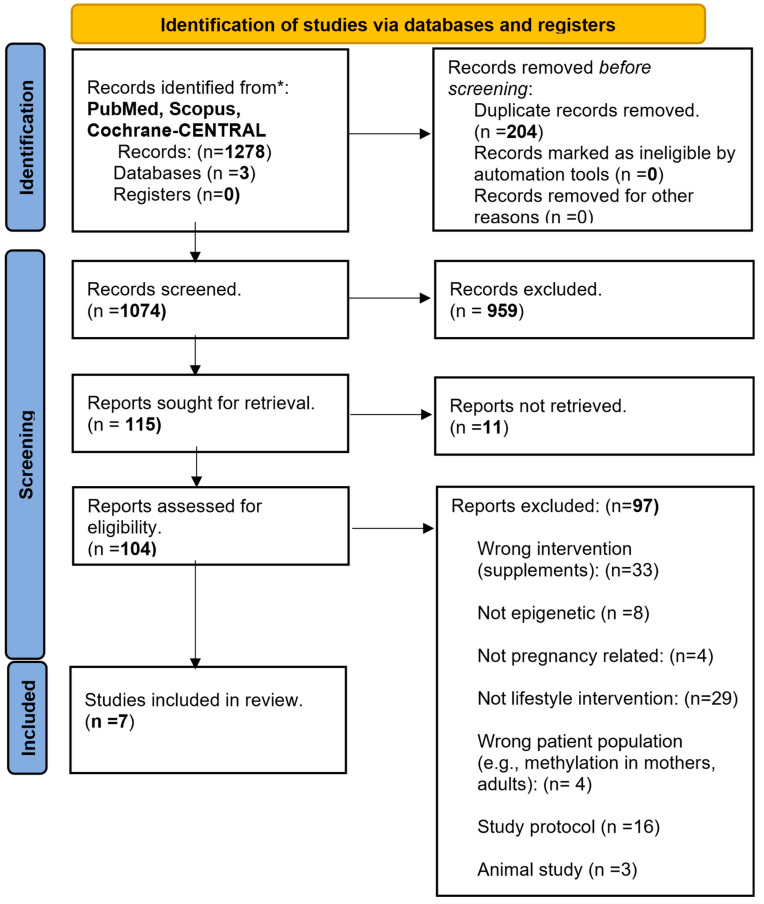
PRISMA flowchart summarizing the steps of the article identification, screening, and inclusion processes. Three databases (PubMed, Scopus and Cochrane-CENTRAL) were searched using the same criteria resulting in 1278 articles. After the exclusion of duplicates (*n* = 204), irrelevant (*n* = 1056), and non-retrievable articles (*n* = 11), seven articles fulfilled the inclusion criteria.

The search of three databases (PubMed, Scopus, and Cochrane-CENTRAL) on 8 July 2023 identified 1278 articles. After the removal of duplicates, animal studies, cohorts, observational studies, studies with wrong interventions (e.g., supplements), patient populations, or outcomes, seven RCTs [44,45,46,47,48,49,50] fulfilled the inclusion criteria and were finally included in the systematic review. Mendeley was used in the selection and elimination process. The flowchart of the article inclusion process is depicted in Figure 1.

### 3.2. Study Characteristics

All seven studies included in this review were RCTs. In total 1765 mother–child pairs were assessed. All studies were published during the last five years (2018–2023) in Ireland [44,46,50], the UK [45], Denmark [48], Australia [49], and Argentina [47]. The participants in all seven RCTs were mothers with a known pathology or risk factor (GDM, increased BMI, and obesity of previous birth of a macrosomic baby).

The intervention in most studies was dietary advice alone or in combination with exercise advice, given at certain timepoints in pregnancy (mostly during or after the second trimester) and sometimes repeated later in gestation, while control subjects received standard care. One study examined the effect of extra virgin olive oil (EVOO) consumption in pregnancy [47]. Study characteristics are summarized in Table 2 below.

### 3.3. Risk Assessment of Included Studies

**Figure 2:**
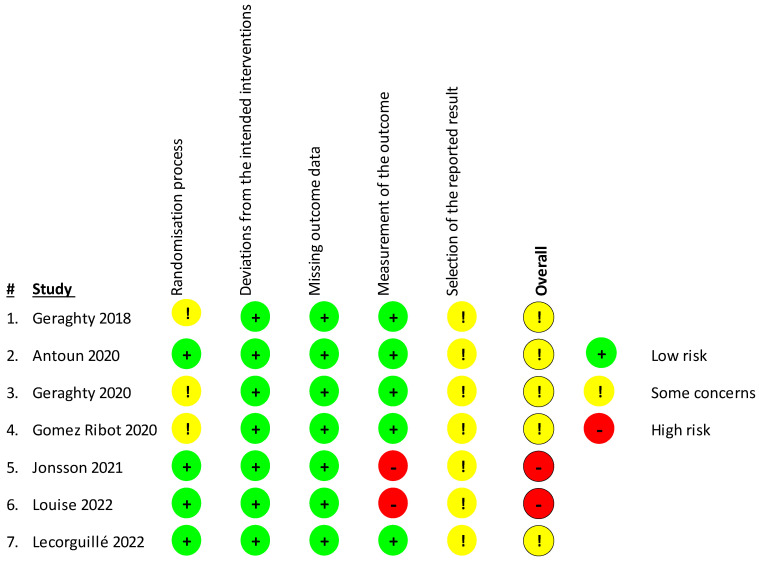
Risk of bias in the included studies was assessed using the Revised Cochrane Risk-Of-Bias Tool for Randomized Trials (RoB 2) investigating bias in the randomization process, bias due to deviations from the intended interventions or missing outcome data, and bias in the measurement of the outcome or selection of the reported result. Studies were considered as having high (red sign) or low risk of bias (green sign) or some concerns depending on the number of individual risk parameters in each of the sections. Table displays studies of Geraghty et al., 2018 [44], Antoun et al., 2020 [45], Geraghty et al., 2020 [46], Gomez Ribot et al., [47], Jonsson et al., 2021 [48], Louise et al., 2022 [49], Lecorguille et al, 2022 [50].

For the assessment of bias of the included studies, the Revised Cochrane Risk-Of-Bias Tool for Randomized Trials (RoB 2) [43] was used (Figure 2). In total, four studies presented some concerns and two [48,49] a high risk of bias as they used a probably non-appropriate methylation analysis tool that covers only a limited amount of CpG sites in the genomeso there may be some differential methylation that went undetected. Concern arose due to possible bias in the selection of the reported result, as many of the studies did not have a study protocol published beforehand to review. Furthermore, some studies [44,46,47] presented some concerns in the randomization process, having some issues with the participant sample sizes.

### 3.4. Synthesis of Results

The synthesis of results is depicted in Table 3, and outcomes and results of the included studies are listed in Table 4 below.

Geraghty et al. [44] examined the effect of advice about healthy eating and a low-glycemic-index diet on offspring methylation at birth [44] and five years of age [46]. The results of their first study indicated a difference in methylation between the intervention and control group where genes affected by a low glycemic index diet were affiliated to cardiac and immune functioning. Decreased average methylation was found in 927 out of the 1000 top differentially methylated probes in the intervention versus control group. In general, the intervention was found to have a subtle influence on neonatal methylome, but the results were weak and unable to be replicated.

Geraghty et al. [46] also studied the effect of the same intervention on the child’s DNAm at five years of age, using the child’s saliva. When a linear regression analysis was conducted, no significant differentially methylated probes between the intervention and control group were found (*p* < 0.05). Thus, no evidence of a lasting effect of the intervention on the methylation status at five years was found.

Antoun et al. [45] assessed physical activity along with a low glycemic index and low saturated fat diet in obese women. This RCT concluded that there were no significant changes in methylation associated with the intervention (FDR ≤ 0.05). However, maternal GDM, and fasting, 1 h, and 2 h plasma glucose levels were associated with methylation in cord blood and differentially methylated CpGs related to cell signaling and transcriptional regulation. Maternal lifestyle intervention in this study was found to attenuate GDM, 1 h and 2 h related methylation, as the effect sizes of the GDM and the 1 h glucose-associated dmCpGs in the intervention versus control arm were 87% and 77% smaller, respectively.

The study of Gomez Ribot et al. [47] was the only one to examine epigenetic modifications in terms of the expression of miRNAs. This RCT studied the effect of three tablespoons of extra virgin olive oil supplementation (EVOO) in pregnant women with GDM on the expression of miR-130a and miR-518d that regulate PPARγ and PPARα (Peroxisome proliferator-activated receptor), respectively, in the placenta. The EVOO diet regulated only miR-518d expression and PPARα levels in the placenta. Normally, miR-518d levels are high in the placentas of women with GDM, and consequently levels of PPARα are low. Women with GDM in the control group had increased miR-518d expression (*p* = 0.003), which was reduced in the intervention group (*p* = 0.009). Thus, the intervention achieved a mild alteration in the epigenetic environment related to GDM in the placenta.

Jönsson et al. [48] studied the effect of both diet and physical activity. Here, authors were seeking a relationship between a Mediterranean-style diet plus increased physical activity (11,000 steps/day) and methylation in the offspring of mothers with obesity. Differential DNAm was found at 379 sites in cord blood, affecting 370 genes in the intervention group (49% of them hypermethylated). Genes were associated with adiposity, obesity, type 2 diabetes, and birth weight. Also, these sites were associated with histone modifications and active chromatin state (H3K4me1), and hence greater gene expression.

The study by Louise et al. [49] examined gene-specific methylation along with genome-wide methylation. This study examined the effect of a balanced diet of low saturated fat, increased fruit and vegetable consumption, and achievable goals in physical activity on whole genome methylation and specific genes related to obesity, metabolism, and growth (IGF2, RXRA, PPARGC1A, and MEST) in the cord blood of children of obese mothers. No association of the intervention to differentially methylated probes in cord blood was found either genome-wide or in candidate genes (PPARGC1A, IGF2, RXRA, or MEST).

The last study [50] investigated the connection of maternal glycemic/insulinemic status and a dietary and exercise intervention during pregnancy with the offspring’s DNAm on cord blood. Maternal insulin concentrations, insulin resistance, β-cell function, and insulin sensitivity were associated with moderate changes in the methylation of CpGs. However, the maternal lifestyle intervention was found to have no effect on DNAm and associations from previous EWAS were not able to be replicated.

## 4. Discussion

### 4.1. Principal Findings

The aim of this systematic review was to assess whether interventions regarding the maternal diet and physical activity during pregnancy can lead to epigenetic alterations in the offspring. These studies suggest that the intervention can lead to either subtle differential methylation or alterations in the pathology-related methylation and that it may have an impact on the offspring’s body composition. Two studies reported significant differences in methylation among control and intervention groups [44,48], which were associated with somatometric effects on the offspring (reduced thigh circumference [44] and more lean mass [48]). Other studies suggested that the intervention altered the GDM-/glucose concentration-related methylation [45], minimizing the detrimental effect of maternal 1 h glucose concentration on the epigenome of the offspring, or that the intervention decreased the elevated GDM-related miR-518d levels [47] contributing to a less inflammatory environment. Two studies were not able to locate statistically significant differences in DNA methylation between the two groups [49,50], while one [46] could not detect a long-lasting effect of the intervention on the methylation at 5 years of age.

### 4.2. Comparison with Existing Literature

Maternal dysglycemia in pregnancy has been associated with long-term risk for the health of the offspring, and this is considered to be mediated by epigenetic modifications. However, recent cohort studies and meta-analyses only weakly associate maternal dysglycemia with DNAm in the offspring [51,52,53,54] finding that the former influence the latter in only a few loci so far.

There is also limited literature considering the effects of lifestyle interventions during pregnancy on the epigenome of the offspring. A recent systematic review [55] concluded there was a possible connection of such an intervention to an effect on DNAm and miRNA expression in cord blood and placenta.

Environmental factors, including GDM, can influence placental epigenetics, which can cause long-term problems in the health of the offspring. GDM, in particular, is linked with methylation changes in cord blood and the placenta [56].

Studies on the methylation of the specific genes investigated (MEST, RXRA, IGF2, and PPARGC1A) in cord blood have linked methylation of the RXRA promoter with adiposity at six and nine years of age and carbohydrate consumption during pregnancy [57]. The MEST gene was significantly less methylated in cord blood and the placenta of pregnant women with GDM rather than healthy women [58]. High blood sugar can change the methylation status of the PPARGC1A gene too, which can lead to obesity and brown adipose tissue problems in the long run [59].

Finally, physical activity during pregnancy was previously related to lower birth weight and PLAGL1 methylation [60]. Very intense physical activity can lead to very low birth weight but working out for a few minutes can impact IGF1-IGF2 levels [61,62], glucose [63], or hormones [64], lessening the chances of a macrosomic infant, and minimizing the effects of hyperglycemia or GDM which were discussed in this review. Only one session of physical activity can reduce methylation levels in promoters of genes such as PPARGC1A and increase their expression in non-pregnant patients [65]. This effect is similar to EVOO consumption in pregnancy [47].

### 4.3. Interpretation of Results

The effect of intervention reported in studies [44,48] can be seen as beneficial as it led to differential methylation that is linked with positive phenotypic results in the offspring such as reduced thigh circumference [44] and more lean mass [48]. Additionally, the intervention appears to minimize the negative epigenetic effects of maternal hyperglycemia on the offspring [45] and contribute to a less inflammatory environment in the placenta via mRNA production [47]. In the long term, DNA methylation patterns related to the intervention are associated with changes in weight centiles at five years of age [46].

Studies that did not report a significant effect of the intervention [46,49,50] supported their findings by using all available data analysis approaches and attributed their discordance to existing literature to diverse sample sizes, inconsistent findings from previous articles, and different data processing methods among studies.

High postprandial glucose levels, such as those seen in participants of the included studies, may have an epigenetic impact on the offspring [45]. The low glycemic index diet, apart from any other effect examined here, is attributed to lower postprandial glucose levels and fewer blood glucose spikes, averting such metabolic and epigenetic responses.

There is no evidence suggesting that a dietary intervention during pregnancy can have a negative impact on the epigenome, or the general health, of the offspring. The outcomes vary from neutral to positive with most studies stating positive effects of the intervention.

### 4.4. Strengths and Limitations

#### 4.4.1. Strengths and Limitations of Included Studies

All studies were RCTs and some of them [46,49] used only data gathered by trained staff and were not relying on self-reported measures like others [48]. Most of the studies used cutting-edge technology to assess the methylation [Illumina Infinum MethylationEPIC BeadChip Array, (HM850K)] [44,45,46,50], while some of them used some previous versions of the tool [48,49]. The included studies used several models to identify associations between the results and factors that may have a biological impact on the methylome but were irrelevant to the intervention (e.g., maternal age, pre-pregnancy BMI, gestational weight gain, breastfeeding, offspring sex, etc.) in order to eliminate confounders and have reliable data.

The studies had some limitations too. Firstly, while assessing the risk of bias, six out of seven studies prompted some concerns since no study protocols were available to review whether the actions carried out were those that were intended. The studies of Jönsson et al. [48] and Louise et al. [49] exhibited a high risk of bias due to the use of a more dated tool for the measurement of methylation, Illumina 450Κ array (Illumina, San Diego, CA, USA). Illumina 450K array covers only 1.7% of CpG sites in the genome [66], so there may be some differential methylation that went undetected.

Secondly, cord blood that was used by most of the studies contains different cell types that could confound the results [44,45,49] and the functional importance of methylation is unknown as epigenetic patterns are different among tissues.

#### 4.4.2. Strengths and Limitations of the Review

While no restrictions were set during the database search, the most important limitation of this review is the heterogeneity of interventions in type (diet/physical activity) or intensity, the women who were randomized, the tools that were used, the tissues, and the types of epigenetic modifications analyzed. The studies included pregnant women with BMI ≥ 30 kg/m^2^ [48], BMI ≥ 25 kg/m^2^ [49], or BMI ≥ 25 kg/m^2^ and ≤39.9 kg/m^2^ [50], obese women with GDM [45], pregnant women with GDM [47], or women who previously had a macrosomic baby [44,46].

Heterogeneity in the studied epigenetic modifications may be the most important limitation. Even though investigating different effects of the interventions may provide a variety of information about several outcomes, the studies used in this review were not able to provide enough information to have a statistically significant result for each intervention. Only one study investigated the effect of the intervention in miRNA expression [49], while two articles had remotely studied some histone modifications along with DNA methylation [44,45]. However, even studies that examined the same epigenetic modification, i.e., DNAm, did not conclude the same methylated sites, so a statistical analysis of the results was impossible. Meanwhile, environmental conditions affect different tissues and histone modifications differently, and miRNA expression may be a better epigenetic indicator of the altered maternal environment caused by the intervention [38,40,44].

The review also included the study of Jönsson et al. [48] and Louise et al. [49] which scored with a high risk of bias in RoB 2. Moreover, only studies that randomized high-risk women (increased BMI, GDM, etc.) were able to be found. No study examined the effect of a lifestyle intervention on the general population. And, lastly, the studies did not examine whether the methylation patterns remained after some time and therefore whether they are permanent traits, apart from one that found no evidence of the lasting effect of the intervention [46].

Considering the limitations of both individual studies and this systematic review, more studies should follow and consider more possible confounders and adjust their calculations to them, where epigenetic modifications should be primary outcome and not just one aspect of the research.

Conclusively, the systematic search resulted in a few, heterogenous studies, with different interventions, outcomes, and results. However, this could be considered an advantage as it provides diversity. Even though, in the majority of the included articles, authors were unable to find statistically significant methylation due to the intervention, the result was ambiguous due to heterogeneity. This is a step towards the correlation of the intrauterine environment with epigenetic changes in the offspring in terms of differential methylation (hyper- or hypomethylation). However, the biological role of such methylation, if there is any, as well as its permanence are still mostly unknown and in the hands of further studies to discover. Thus, more RCTs focused on the epigenetic effect of a lifestyle intervention in pregnancy must follow, with large and common sample sizes, populations, interventions, outcomes, and tools used, so a statistical analysis of the results can be conducted. Paternal lifestyle contributions to offspring’s epigenome should also be studied, as well as the epigenetic modifications during the pre-conceptional period, which plays a major role in the human epigenome [67,68].

## 5. Conclusions

This review concluded that an intervention in maternal lifestyle during pregnancy with a low-glycemic-index diet or increase in physical activity can, even slightly, impact DNAm or miRNA expression in cord blood and the placenta in women with a previous condition (GDM, obesity, etc.). Secondly, the intervention had an impact on the offspring’s body composition and DNAm in regions related to obesity, diabetes, adiposity, and birth weight. However, due to the small number and heterogeneity of the included studies and the non-specified biological role of differential methylation, there is a need for more studies that will methodically examine the impact of a maternal and paternal lifestyle intervention before and after conception on the offspring’s epigenome. This will help to elucidate the ways conditions like cancer, obesity, diabetes, or other metabolic diseases emerge and become established.

## Figures and Tables

**Table 1:** PICO table about the included studies. Contains information about the studies’ author, country of origin, participants, type of intervention, period of intervention, comparator/control, main characteristics/confounders, and outcomes.

	Author	Country	Participants	Intervention	Intervention Period	Control	Characteristics	Outcomes
1.	Geraghty (2018) [44] RCT	Ireland	*n* = 60 sex-matched neonates (30 intervention 30 control from the ROLO study) ROLO study: women ≥ 18 who previously gave birth to a macrosomic baby	*n* = 30 Dietary advice about healthy eating and a low glycemic index diet given in second trimester.	Three times in pregnancy (18, 28 and 34 weeks).	*n* = 30 No specific dietary advice	Chip position Offspring sex Gestational age	Mean difference in Glycemic Index before and after the intervention. Association of maternal and neonatal factors (weight, BMI) to methylation Genome-wide DNA methylation in cord blood and cord serum Differentially methylated probes in intervention vs. control group Functional clusters of methylated probes Cell type analysis
2.	Antoun (2020) [45] RCT	UK	*n* = 557 (buffy coat samples from) pregnant women from the UPBEAT RCT with BMI ≥ 30 pre pregnancy randomized between 15 + 0 and 18 + 6 to standard care or intervention.	*n* = 263 Low glycemic index diet, low saturated fat, increased physical activity.	Eight hourly sessions once/week for 8 weeks	*n* = 294 Treated using standard protocols. (159 of them were diagnosed with GDM)	Neonate sex Maternal ethnicity/parity/smoking/BMI/age Predicted values for white blood cells Nucleated red blood cell composition	Cord blood DNA methylation in infants of obese mothers who developed GDM and those who did not. GDM/dysglycemia-associated dmCpGs in cord blood Modification of the (previous) impact of GDM in offspring’s methylation caused by the intervention. GDM associated dmCpGs relation to histone modifications. GDM-associated dmCpGs relation to (functional) networks Sexual dimorphism in GDM-associated methylation Influence of genetic variation on the GDM-associated dmCpGs
3.	Geraghty (2020) [46] RCT	Ireland	*n* = 63 5 year-olds from the ROLO study	*n* = 31 Dietary advice about healthy eating and a low glycemic index diet given in second trimester.	Three times in pregnancy (18, 28 and 34 weeks).	*n* = 32 No specific dietary advice	Chip position Offspring sex	Lasting effect of the intervention to the methylation status in 5 years. DNA methylation in saliva samples at 5 years of age Functional clusters of methylated probes Association of child’s body composition and adiposity to methylation at 5 years of age Adiposity and body composition at birth, six months, 2 and 5 years of age
4.	Gomez Ribot (2020) [47] RCT	Argentina	*n* = 60 Pregnant women with singleton pregnancy and GDM at 24–28 wks divided in three groups: Control, GDM and GDM-EVOO. Results available for: Control (*n* = 15), GDM (*n* = 15), GDM-EVOO (*n* = 15)	Three tablespoons of uncooked EVOO daily (36 g/day) within the meals. All women were given dietary advice to follow: 2100–2400 Kcal/day; carbohydrates 48–50%, proteins 18–20% and lipids 30–32%.	Enrollment at 24–28 wks.	None to one tablespoon of EVOO daily (0–12 g/day). All women were given dietary advice to follow: 2100–2400 Kcal/day; carbohydrates 48–50%, proteins 18–20% and lipids 30–32%.	Not stated	PPARα and PPARγ levels and prooxidant/proinflammatory markers in placenta (NO, TNF, IL-1β, SOD expression, gelatinase activity of matrix metalloproteinases) Maternal weight gain, neonatal/placental weight, changes in maternal metabolic control, triglyceridemia, expression of miRNAs that target PPARα and PPARγ (miR-518d and miR-130a)
5.	Jönsson (2021) [48] RCT	Denmark	*n* = 208 DNA samples from obese pregnant women (BMI ≥ 30 kg/m^2^) in TOP study	Two groups: Physical activity (assessed with pedometer) + diet (*n* = 76) Physical activity (assessed with pedometer) (*n* = 59) Women of both groups were recommended to walk 11,000 steps/day, maintain a low-fat Mediterranean-style diet of 1200–1675 kcal and keep GWG to ≤5 kg.	Enrollment at 11–14 weeks. Only the PA + D group received follow-up appointments every 2 weeks	*n* = 73 Standard of care + recommendation of keeping a low-fat Mediterranean-style diet of 1200–1675 kcal and limit GWG to ≤5 kg.	Maternal age Smoking Gestational age GWG Pre-pregnancy BMI Offspring sex	Differential cord blood DNA methylation between the two groups Association of DNA methylation to lean mass at birth and growth. Body composition at birth, 9 and 36 months of age Association of GWG with cord blood DNA methylation Involvement of methylated sites to metabolic processes Association of methylated sites to histone modifications DNA methylation assessment also in muscles and adipose tissue
6.	Louise (2022) [49] RCT	Australia	*n* = 645 randomly selected and balanced between the two groups cord blood samples from participants of the LIMIT RCT. Pregnant women with BMI ≥ 25 kg/m^2^	*n* = 325 Dietary and lifestyle advice (healthy alternatives to sugar and achievable goals in physical activity)	Enrollment at 10–20 weeks and follow-up sessions with the dietitian or research assistants at 22, 24, 28, 32, 36 weeks	*n* = 320 Standard care. No specific advice on diet or activity.	Maternal BMI/age/parity/ Smoking status Offspring sex Study center	Genome-wide differential methylation between lifestyle and control group Intervention-/maternal BMI-associated methylation in candidate genes (PPARGC1A, IGF2, RXRA, or MEST)
7.	Lecorguillé (2022) [50] RCT	Ireland	*n* = 172 Cord blood samples from participants of the PEARS RCT. Singleton pregnancy women BMI ≥ 25 kg/m^2^ and ≤39.9 kg/m^2^	*n* = 96 Dietary advice about a low GI diet and recommendation on portion sizes of carbohydrates + moderate exercise of 30 min for 5–7 days/week. Smartphone app and emails every 2 weeks.	Enrollment at 10–15 weeks. Follow-up sessions for the intervention group at 28 and 34 weeks.	Standard care without consistent dietary, exercise of gestational weight gain advice.	Child sex, gestational age at birth Batch effect Cell type proportions Parity Maternal education/smoking/ethnicity/age/BMI Birthweight.	Connection of maternal prenatal glycemia and insulin status to methylation patterns in cord blood of offspring. Effect of an intervention with dietary advice and moderate exercise in women with between BMI ≥ 25 kg/m^2^ and ≤39.9 kg/m^2^ pregnancy to epigenetic change in the offspring.

**Table 2:** Summary of study characteristics including authors’ names, country of origin, name of the original study, type of intervention, outcome, tissue of interest, and tools used.

	Authors Name	Country	Study	Intervention	Outcome	Tissue of Interest	Tool Used
1.	Geraghty et al., 2018 [44]	Ireland	ROLO	Dietary advice	Epigenome-wide DNA methylation of offspring at birth	Cord blood	Illumina Infinum MethylationEPIC BeadChip array (HM850K)
2.	Antoun et al., 2020 [45]	UK	UPBEAT	Dietary advice + physical activity	Epigenome-wide DNA methylation of offspring at birth	Cord blood	Infinium Human MethylationEPIC BeadChip array (HM850K)
3.	Geraghty et al., 2020 [46]	Ireland	ROLO	Dietary advice	Epigenome-wide DNA methylation of offspring at 5 years of age	Saliva	Illumina MethylationEPIC array (HM850K)
4.	Gomez Ribot et al., 2020 [47]	Argentina	[nameless]	Three tablespoons of EVOO	miRNA expression	Placenta	TaqMan MicroRNA reverse transcription kit
5.	Jönsson et al., 2021 [48]	Denmark	TOP	Two groups: Physical activity + diet Physical activity	Epigenome-wide DNA methylation of offspring at birth	Cord blood	Illumina Infinium Human Methylation450 BeadChip array
6.	Louise et al., 2022 [49]	Australia	LIMIT	Dietary advice + physical activity	Epigenome-wide DNA methylation of offspring + methylation at specific genes at birth	Cord blood	Illumina Infinium Human Methylation 450 BeadChip array
7.	Lecorguillé et al., 2022 [50]	Ireland	PEARS	Dietary advice + moderate exercise	Epigenome-wide DNA methylation of offspring at birth	Cord blood	Illumina Infinium Human MethylationEPIC array (HM850K)

**Table 3:** Synthesis of studies. Studies with significant differences between intervention and control groups are depicted in green color, those where the intervention altered the pathology related DNA methylation or miRNA expression are shown in yellow, and studies with statistically non-significant differences between intervention and control groups are seen in red.

Results	Studies
Statistically significant differences between intervention-control groups	Geraghty et al., 2018 [44] Jönsson et al., 2021 [48]
Intervention altered the pathology-related methylation/miRNA expression	Antoun et al., 2020 [45] Gomez Ribot et al., 2020 [47]
Statistically non-significant differences between intervention-control groups	Geraghty et al., 2020 [46] Louise et al., 2022 [49] Lecorguillé et al., 2022 [50]

**Table 4:** Outcome measures and results of the included studies.

	Study	Outcome Measures	Findings
1.	Geraghty et al., 2018 [44] (RCT)	Cord blood genome-wide DNAm changes in offspring of mothers participating in the ROLO study of a low-glycemic-index diet intervention in pregnancy.	Subtle differences in DNAm in cord blood and cord serum. Differentially methylated probes in intervention vs. control group. Genes affected by low glycemic index diet are related to cardiac and immune functioning. Maternal BMI/birth weight irrelevant to offspring methylome.
2.	Antoun et al., 2020 [45] (RCT)	Cord blood DNAm in offspring of obese mothers (BMI ≥ 30) with dysglycemia at 24–28 weeks and effect of a dietary and physical activity intervention to it. (Part of the UPBEAT RCT)	DNAm in cord blood. GDM, FPG, 1 h, and 2 h PG levels were associated with dmCpGs related to cell signaling and transcriptional regulation. Maternal lifestyle intervention attenuated GDM, 1 h and 2 h related methylation. Differences in cord blood DNAm due to maternal dysglycemia between offspring sexes.
3.	Geraghty et al., 2020 [46] (RCT)	Genome-wide DNAm in saliva samples of 5-year-old children whose mothers participated in the ROLO study taking dietary advice about healthy eating and a low glycemic index diet in the second trimester. Adiposity and body composition at birth, six months, 2, and 5 years of age.	Maternal factors (nutrition) during pregnancy, child’s weight at birth and gestational age, and child’s body composition or adiposity at 5 years are not associated with methylation status at 5 years but change in weight centiles and BMI at six months were associated with some variation of the DNAm. No evidence of lasting effect of the intervention to the methylation status in 5 years. GI of mother in the 1st trimester is related to some variation in DNAm of the child. Growth patterns may be linked with the methylome in childhood. Functional clusters of methylated probes are related to insulin signaling/resistance.
4.	Gomez Ribot et al., 2020 [47] (RCT)	Expression of miR-518d and miR-130a (miRNAs that target PPARα and PPARγ) in placentas of women taking three tablespoons of extra virgin olive oil at 24–28 wks of pregnancy.	GDM group gained more weight unlike the intervention group. EVOO diet prevented increase of triglyceridemia and weight gain in the GDM-EVOO group compared to the GDM vs. control. EVOO diet induced anti-inflammatory effects in the placenta (did not regulate miRNA-130a expression or PPARγ levels but did regulate miRNA-518d expression and PPARα levels in the placenta)
5.	Jönsson et al., 2021 [48] (RCT)	Genome-wide cord blood DNAm changes in offspring of obese mothers participating in the TOP study, studying the effect of physical activity with or without a diet intervention (low energy/low-fat Mediterranean-style diet) in pregnancy. Association of DNAm to lean mass at birth, growth and body composition at birth, 9 and 36 months of age.	Cord blood DNAm differs between the two groups. Many differentially methylated sites are part of metabolic processes or associated with SNPs related to adiposity, obesity, type 2 diabetes, birth weight, or other disease. Children of mothers from the intervention group have more lean mass (increased abdominal and a trend towards increased total lean mass). Positive association of methylation in cord blood and increased lean mass, partly explained by the hypomethylation of SETD3 which is related to increased muscle mass. Lean mass-associated methylation pattern in cord blood resembles the one on muscles and adipose tissue, both important in metabolic disease.
6.	Louise et al., 2022 [49] (RCT)	Cord blood DNAm and methylation of specific candidate genes in offspring of mothers (BMI ≥ 25 kg/m^2^) who participated in the LIMIT study getting dietary and lifestyle advice (healthy alternatives to sugar and achievable goals in physical activity). The genes assessed were related to obesity, metabolism, adiposity, and growth (PPARGC1A, IGF2, RXRA, or MEST)	Intervention, BMI, or their interaction were not associated with differentially methylated probes in cord blood; BMI was associated with the methylation of five probes in the standard care group. Not reproducible results. No significant differences in methylation associated with intervention or maternal BMI were found in candidate genes (PPARGC1A, IGF2, RXRA, or MEST). Some statistically significant methylated probes were found, but the results are doubtable.
7.	Lecorguillé et al., 2022 [50] (RCT)	Genome-wide DNAm in cord blood of offspring of mothers with BMI ≥ 25 kg/m^2^ and ≤39.9 kg/m^2^ who participated in a RCT of dietary advice and moderate exercise in pregnancy.	Maternal insulin concentrations, insulin resistance, β-cell function, insulin sensitivity, glycemic load, glycemic index were associated with CpGs located near/in RNF214 and PCSK7, SYN3, JARID2, POLR2C, LINC01150, and OCA2 genes, respectively. No effect of the intervention on cord blood DNAm.

## Appendix A

**Table A1:** Search strategy.

Column 1		Column 2		Column 3
Pregnan *		Epigen *		Lifestyle
Gestation *		DNA methylation		Diet *
Periconception*	AND	Methylation	AND	Physical activity
Preconception *		Histone		Stress
Mother		Acetylation		Sedentary
Matern *		Phosphorylation		
Infant		miRNA		
Offspring				

Words within the column were combined with OR and across columns with AND, as indicated. Asterisks were part of the word in the search process.

PubMed on 8/7/2023
  (pregnan* OR gestation OR mother OR matern* OR infant OR offspring OR periconception* OR preconception*) AND (epigen* OR “DNA methylation” OR methylation OR methyl* OR miRNA OR histone OR acetylation OR modification OR phosphorylation) AND (lifestyle OR diet* OR Mediterranean OR food OR nutri* OR metal* OR pollution OR “physical activity” OR sedentary OR stress)
  + setting filters to “Clinical trials” and “Randomized controlled trials” → 562 results
Scopus on 8/7/2023
  TITLE-ABS-KEY (pregnan* AND epigen* AND (lifestyle OR diet* OR “physical activity” OR stress OR pollution) AND trial) AND (LIMIT-TO (SRCTYPE, “j”)) AND (LIMIT-TO (SUBJAREA, “MEDI”) OR LIMIT-TO (SUBJAREA, “BIOC”) OR LIMIT-TO (SUBJAREA, “IMMU”) OR LIMIT-TO (SUBJAREA, “PHAR”) OR LIMIT-TO (SUBJAREA, “NEUR”) OR LIMIT-TO (SUBJAREA, “PSYC”)) AND (LIMIT-TO (LANGUAGE, “English”)) AND (LIMIT-TO (EXACTKEYWORD, “Human”)) → 172 results
Cochrane-CENTRAL in 8/7/2023
  (pregnan* OR gestation OR mother OR matern* OR infant OR offspring) AND (epigen* OR “DNA methylation” OR methylation OR methyl* OR histone) AND (lifestyle OR diet* OR pollution OR “physical activity” OR stress)
  + filters “Trials” and “Clinical answers” → 544 results
In total: 1278 articles

## References

[1] Reijnders I.F., Mulders A.G.M.G.J., van der Windt M., Steegers E.A.P., Steegers-Theunissen R.P.M. (2018). The Impact of Periconceptional Maternal Lifestyle on Clinical Features and Biomarkers of Placental Development and Function: A Systematic Review. Hum. Reprod. Update.

[2] Zerfu T.A., Pinto E., Baye K. (2018). Consumption of Dairy, Fruits and Dark Green Leafy Vegetables Is Associated with Lower Risk of Adverse Pregnancy Outcomes (APO): A Prospective Cohort Study in Rural Ethiopia. Nutr. Diabetes.

[3] Moody L., Chen H., Pan Y.-X. (2017). Early-Life Nutritional Programming of Cognition—The Fundamental Role of Epigenetic Mechanisms in Mediating the Relation between Early-Life Environment and Learning and Memory Process. Adv. Nutr. Int. Rev. J..

[4] Hales C., Barker D. (2013). Type 2 (Non-Insulin-Dependent) Diabetes Mellitus: The Thrifty Phenotype Hypothesis. Int. J. Epidemiol..

[5] Marciniak A., Patro-Małysza J., Kimber-Trojnar Ż., Marciniak B., Oleszczuk J., Leszczyńska-Gorzelak B. (2017). Fetal Programming of the Metabolic Syndrome. Taiwan. J. Obstet. Gynecol..

[6] Marshall M.R., Paneth N., Gerlach J., Mudd L.M., Biery L., Ferguson D.P., Pivarnik J.M. (2018). Differential Methylation of Insulin-like Growth Factor 2 in Offspring of Physically Active Pregnant Women. J. Dev. Orig. Health Dis..

[7] Barker D.J. (1990). The Fetal and Infant Origins of Adult Disease. BMJ.

[8] Barker D.J.P. (2007). The Origins of the Developmental Origins Theory. J. Intern. Med..

[9] Perrone S., Santacroce A., Picardi A., Buonocore G. (2016). Fetal Programming and Early Identification of Newborns at High Risk of Free Radical-Mediated Diseases. World J. Clin. Pediatr..

[10] Franzago M., Fraticelli F., Stuppia L., Vitacolonna E. (2019). Nutrigenetics, Epigenetics and Gestational Diabetes: Consequences in Mother and Child. Epigenetics.

[11] Bianco-Miotto T., Craig J.M., Gasser Y.P., van Dijk S.J., Ozanne S.E. (2017). Epigenetics and DOHaD: From Basics to Birth and Beyond. J. Dev. Orig. Health Dis..

[12] Yang X., Han H., De Carvalho D.D., Lay F.D., Jones P.A., Liang G. (2014). Gene Body Methylation Can Alter Gene Expression and Is a Therapeutic Target in Cancer. Cancer Cell.

[13] OSBORNE-MAJNIK A., FU Q., LANE R.H. (2013). Epigenetic Mechanisms in Fetal Origins of Health and Disease. Clin. Obstet. Gynecol..

[14] Fleming T.P., Eckert J.J., Denisenko O. (2017). The Role of Maternal Nutrition during the Periconceptional Period and Its Effect on Offspring Phenotype. Adv. Exp. Med. Biol..

[15] Rivera R.M., Ross J.W. (2013). Epigenetics in Fertilization and Preimplantation Embryo Development. Prog. Biophys. Mol. Biol..

[16] Neidhart M. (2016). DNA Methylation—Introduction. DNA Methylation and Complex Human Disease.

[17] Cutter A.R., Hayes J.J. (2015). A Brief Review of Nucleosome Structure. FEBS Lett..

[18] Wilczynska A., Bushell M. (2014). The Complexity of MiRNA-Mediated Repression. Cell Death Differ..

[19] Chang G., Mouillet J.F., Mishima T., Chu T., Sadovsky E., Coyne C.B., Parks W.T., Surti U., Sadovsky Y. (2017). Expression and Trafficking of Placental MicroRNAs at the Feto-Maternal Interface. FASEB J..

[20] Zhu W., Shen Y., Liu J., Fei X., Zhang Z., Li M., Chen X., Xu J., Zhu Q., Zhou W. (2020). Epigenetic Alternations of MicroRNAs and DNA Methylation Contribute to Gestational Diabetes Mellitus. J. Cell. Mol. Med..

[21] Chen P., Piaggi P., Traurig M., Bogardus C., Knowler W.C., Baier L.J., Hanson R.L. (2017). Differential Methylation of Genes in Individuals Exposed to Maternal Diabetes in Utero. Diabetologia.

[22] Awamleh Z., Butcher D.T., Hanley A., Retnakaran R., Haertle L., Haaf T., Hamilton J., Weksberg R. (2021). Exposure to Gestational Diabetes Mellitus (GDM) Alters DNA Methylation in Placenta and Fetal Cord Blood. Diabetes Res. Clin. Pract..

[23] He J., Liu K., Hou X., Lu J. (2021). Comprehensive Analysis of DNA Methylation and Gene Expression Profiles in Gestational Diabetes Mellitus. Medicine.

[24] Gemma C., Sookoian S., Alvariñas J., García S.I., Quintana L., Kanevsky D., González C.D., Pirola C.J. (2009). Maternal Pregestational BMI Is Associated with Methylation of thePPARGC1APromoter in Newborns. Obesity.

[25] Oelsner K.T., Guo Y., To S.B.-C., Non A.L., Barkin S.L. (2017). Maternal BMI as a Predictor of Methylation of Obesity-Related Genes in Saliva Samples from Preschool-Age Hispanic Children At-Risk for Obesity. BMC Genom..

[26] Nogues P., Dos Santos E., Jammes H., Berveiller P., Arnould L., Vialard F., Dieudonné M.-N. (2019). Maternal Obesity Influences Expression and DNA Methylation of the Adiponectin and Leptin Systems in Human Third-Trimester Placenta. Clin. Epigenetics.

[27] Berglind D., Müller P., Willmer M., Sinha I., Tynelius P., Näslund E., Dahlman-Wright K., Rasmussen F. (2015). Differential Methylation in Inflammation and Type 2 Diabetes Genes in Siblings Born before and after Maternal Bariatric Surgery. Obesity.

[28] Guenard F., Deshaies Y., Cianflone K., Kral J.G., Marceau P., Vohl M.-C. (2013). Differential Methylation in Glucoregulatory Genes of Offspring Born before vs. after Maternal Gastrointestinal Bypass Surgery. Proc. Natl. Acad. Sci. USA.

[29] Robinson S.L., Mumford S.L., Guan W., Zeng X., Kim K., Radoc J.G., Trinh M.-H., Flannagan K., Schisterman E.F., Yeung E. (2019). Maternal Fatty Acid Concentrations and Newborn DNA Methylation. Am. J. Clin. Nutr..

[30] Fan C., Fu H., Dong H., Lu Y., Lu Y., Qi K. (2016). Maternal N-3 Polyunsaturated Fatty Acid Deprivation during Pregnancy and Lactation Affects Neurogenesis and Apoptosis in Adult Offspring: Associated with DNA Methylation of Brain-Derived Neurotrophic Factor Transcripts. Nutr. Res..

[31] Cinquina V., Calvigioni D., Farlik M., Halbritter F., Fife-Gernedl V., Shirran S.L., Fuszard M.A., Botting C.H., Poullet P., Piscitelli F. (2019). Life-Long Epigenetic Programming of Cortical Architecture by Maternal “Western” Diet during Pregnancy. Mol. Psychiatry.

[32] House J.A., Mendez M.A., Maguire R.L., Gonzalez-Nahm S., Huang Z., Daniels J.L., Murphy S.K., Fuemmeler B.F., Wright F.A., Hoyo C. (2018). Periconceptional Maternal Mediterranean Diet Is Associated with Favorable Offspring Behaviors and Altered CpG Meth-ylation of Imprinted Genes. Front. Cell Dev. Biol..

[33] Gonzalez-Nahm S., Mendez M., Robinson W., Murphy S.K., Hoyo C., Hogan V., Rowley D. (2017). Low Maternal Adherence to a Mediterranean Diet Is Associated with Increase in Methylation at the MEG3-IG Differentially Methylated Region in Female Infants. Environ. Epigenetics.

[34] Küpers L.K., Fernández-Barrés S., Nounu A., Friedman C., Fore R., Mancano G., Dabelea D., Rifas-Shiman S.L., Mulder R.H., Oken E. (2022). Maternal Mediterranean Diet in Pregnancy and Newborn DNA Methylation: A Meta-Analysis in the PACE Consortium. Epigenetics.

[35] Kingsley S.L., Eliot M.N., Whitsel E.A., Huang Y.-T., Kelsey K.T., Marsit C.J., Wellenius G.A. (2016). Maternal Residential Proximity to Major Roadways, Birth Weight, and Placental DNA Methylation. Environ. Int..

[36] Ladd-Acosta C., Feinberg J.I., Brown S.C., Lurmann F.W., Croen L.A., Hertz-Picciotto I., Newschaffer C.J., Feinberg A.P., Fallin M.D., Volk H.E. (2019). Epigenetic Marks of Prenatal Air Pollution Exposure Found in Multiple Tissues Relevant for Child Health. Environ. Int..

[37] Brooks S.A., Fry R.C. (2017). Cadmium Inhibits Placental Trophoblast Cell Migration via MiRNA Regulation of the Transforming Growth Factor Beta (TGF-β) Pathway. Food Chem. Toxicol..

[38] Rager J.E., Bailey K.A., Smeester L., Miller S.K., Parker J.S., Drobná Z., Currier J.M., Douillet C., Olshan A.F., Rubio-Andrade M. (2014). Prenatal Arsenic Exposure and the Epigenome: Altered MicroRNAs Associated with Innate and Adaptive Immune Signaling in Newborn Cord Blood. Environ. Mol. Mutagen..

[39] Sood S., Shekhar S., Santosh W. (2017). Dimorphic Placental Stress: A Repercussion of Interaction between Endocrine Disrupting Chemicals (EDCs) and Fetal Sex. Med. Hypotheses.

[40] Gillet V., Hunting D.J., Takser L. (2016). Turing Revisited: Decoding the MicroRNA Messages in Brain Extracellular Vesicles for Early Detection of Neurodevelopmental Disorders. Curr. Environ. Health Rep..

[41] Hompes T., Izzi B., Gellens E., Morreels M., Fieuws S., Pexsters A., Schops G., Dom M., Van Bree R., Freson K. (2013). Investigating the Influence of Maternal Cortisol and Emotional State during Pregnancy on the DNA Methylation Status of the Glucocorticoid Receptor Gene (NR3C1) Promoter Region in Cord Blood. J. Psychiatr. Res..

[42] Oberlander T.F., Weinberg J., Papsdorf M., Grunau R., Misri S., Devlin A.M. (2008). Prenatal Exposure to Maternal Depression, Neonatal Methylation of Human Glucocorticoid Receptor Gene (NR3C1) and Infant Cortisol Stress Responses. Epigenetics.

[43] Sterne J.A., Savović J., Page M.J., Elbers R.G., Blencowe N.S., Boutron I., Cates C.J., Cheng H.Y., Corbett M.S., Eldridge S.M. (2019). RoB 2: A revised tool for assessing risk of bias in randomised trials. BMJ.

[44] Geraghty A., Sexton-Oates A., O’Brien E., Alberdi G., Fransquet P., Saffery R., McAuliffe F. (2018). A Low Glycaemic Index Diet in Pregnancy Induces DNA Methylation Variation in Blood of Newborns: Results from the ROLO Randomised Controlled Trial. Nutrients.

[45] Antoun E., Kitaba N.T., Titcombe P., Dalrymple K.V., Garratt E.S., Barton S.J., Murray R., Seed P.T., Holbrook J.D., Kobor M.S. (2020). Maternal Dysglycaemia, Changes in the Infant’s Epigenome Modified with a Diet and Physical Activity Intervention in Pregnancy: Secondary Analysis of a Randomised Control Trial. PLoS Med..

[46] Geraghty A.A., Sexton-Oates A., O’Brien E.C., Saffery R., McAuliffe F.M. (2020). Epigenetic Patterns in Five-Year-Old Children Exposed to a Low Glycemic Index Dietary Intervention during Pregnancy: Results from the ROLO Kids Study. Nutrients.

[47] Gomez Ribot D., Diaz E., Fazio M.V., Gómez H.L., Fornes D., Macchi S.B., Gresta C.A., Capobianco E., Jawerbaum A. (2020). An Extra Virgin Olive Oil-Enriched Diet Improves Maternal, Placental and Cord Blood Parameters in GDM Pregnancies. Diabetes/Metab. Res. Rev..

[48] Jönsson J., Renault K.M., García-Calzón S., Perfilyev A., Estampador A.C., Nørgaard K., Lind M.V., Vaag A., Hjort L., Michaelsen K.F. (2021). Lifestyle Intervention in Pregnant Women with Obesity Impacts Cord Blood DNA Methylation, Which Associates with Body Composition in the Offspring. Diabetes.

[49] Louise J., Deussen A.R., Koletzko B., Owens J.A., Saffery R., Dodd J.M. (2022). Effect of an Antenatal Diet and Lifestyle Intervention and Maternal BMI on Cord Blood DNA Methylation in Infants of Overweight and Obese Women: The LIMIT Randomised Controlled Trial. PLoS ONE.

[50] Lecorguillé M., McAuliffe F.M., Twomey P.J., Viljoen K., Mehegan J., Kelleher C.C., Suderman M., Phillips C.M. (2022). Maternal Glycaemic and Insulinemic Status and Newborn DNA Methylation: Findings in Women with Overweight and Obesity. J. Clin. Endocrinol. Metab..

[51] Tobi E.W., Juvinao-Quintero D.L., Ronkainen J., Ott R., Alfano R., Canouil M., Khamis A., Küpers L.K., Lim I.Y., Perron P. (2022). Maternal Glycemic Dysregulation during Pregnancy and Neonatal Blood DNA Methylation: Meta-Analyses of Epigenome-Wide Association Studies. Diabetes Care.

[52] Howe C.G., Cox B., Fore R., Jungius J., Kvist T., Lent S., Miles H.E., Salas L.A., Rifas-Shiman S., Starling A.P. (2020). Maternal Gestational Diabetes Mellitus and Newborn DNA Methylation: Findings from the Pregnancy and Childhood Epigenetics Consortium. Diabetes Care.

[53] Geurtsen M.L., Vincent, Gaillard R., Felix J.F. (2020). Associations of Maternal Early-Pregnancy Blood Glucose and Insulin Concentrations with DNA Methylation in Newborns. Clin. Epigenetics.

[54] Canouil M., Khamis A., Keikkala E., Hummel S., Lobbens S., Bonnefond A., Delahaye F., Tzala E., Mustaniemi S., Vääräsmäki M. (2021). Epigenome-Wide Association Study Reveals Methylation Loci Associated with Offspring Gestational Diabetes Mellitus Exposure and Maternal Methylome. Diabetes Care.

[55] Rasmussen L., Knorr S., Antoniussen C.S., Bruun J.M., Ovesen P.G., Fuglsang J., Kampmann U. (2021). The Impact of Lifestyle, Diet and Physical Activity on Epigenetic Changes in the Offspring—A Systematic Review. Nutrients.

[56] Ruchat S.-M., Houde A.-A., Voisin G., St-Pierre J., Perron P., Baillargeon J.-P., Gaudet D., Hivert M.-F., Brisson D., Bouchard L. (2013). Gestational Diabetes Mellitus Epigenetically Affects Genes Predominantly Involved in Metabolic Diseases. Epigenetics.

[57] Godfrey K.M., Sheppard A., Gluckman P.D., Lillycrop K.A., Burdge G.C., McLean C., Rodford J., Slater-Jefferies J.L., Garratt E., Crozier S.R. (2011). Epigenetic Gene Promoter Methylation at Birth Is Associated with Child’s Later Adiposity. Diabetes.

[58] El Hajj N., Pliushch G., Schneider E., Dittrich M., Muller T., Korenkov M., Aretz M., Zechner U., Lehnen H., Haaf T. (2012). Metabolic Programming of MEST DNA Methylation by Intrauterine Exposure to Gestational Diabetes Mellitus. Diabetes.

[59] Côté S., Gagné-Ouellet V., Guay S.-P., Allard C., Houde A.-A., Perron P., Baillargeon J.-P., Gaudet D., Guérin R., Brisson D. (2016). PPARGC1α Gene DNA Methylation Variations in Human Placenta Mediate the Link between Maternal Hyperglycemia and Leptin Levels in Newborns. Clin. Epigenetics.

[60] McCullough L.E., Mendez M.A., Miller E.E., Murtha A.P., Murphy S.K., Hoyo C. (2015). Associations between Prenatal Physical Activity, Birth Weight, and DNA Methylation at Genomically Imprinted Domains in a Multiethnic Newborn Cohort. Epigenetics.

[61] Hopkins S.A., Baldi J.C., Cutfield W.S., McCowan L., Hofman P.L. (2010). Exercise Training in Pregnancy Reduces Offspring Size without Changes in Maternal Insulin Sensitivity. J. Clin. Endocrinol. Metab..

[62] Hopkins S.A., Baldi J.C., Cutfield W.S., McCowan L., Hofman P.L. (2011). Effects of Exercise Training on Maternal Hormonal Changes in Pregnancy. Clin. Endocrinol..

[63] McMurray R.G., Hackney A.C., Guion W.K., Katz V.L. (1996). Metabolic and Hormonal Responses to Low-Impact Aerobic Dance during Pregnancy. Med. Sci. Sports Exerc..

[64] Bonen A., Campagna P.D., Gilchrist L., Beresford P. (1995). Substrate and Hormonal Responses during Exercise Classes at Selected Stages of Pregnancy. Can. J. Appl. Physiol..

[65] Barrès R., Yan J., Egan B., Treebak J.T., Rasmussen M., Fritz T., Caidahl K., Krook A., O’Gorman D.J., Zierath J.R. (2012). Acute Exercise Remodels Promoter Methylation in Human Skeletal Muscle. Cell Metab..

[66] Sharp G.C., Lawlor D.A., Richmond R.C., Fraser A., Simpkin A., Suderman M., Shihab H.A., Lyttleton O., McArdle W., Ring S.M. (2015). Maternal Pre-Pregnancy BMI and Gestational Weight Gain, Offspring DNA Methylation and Later Offspring Adiposity: Findings from the Avon Longitudinal Study of Parents and Children. Int. J. Epidemiol..

[67] Fleming T.P., Velazquez M.A., Eckert J.J. (2015). Embryos, DOHaD and David Barker. J. Dev. Orig. Health Dis..

[68] Franzago M., Rovere M.L., Franchi P.G., Vitacolonna E., Stuppia L. (2019). Epigenetics and Human Reproduction: The Primary Prevention of the Noncommunicable Diseases. Epigenomics.

